# Host Responses to SARS-CoV-2 with an Emphasis on Cytokines

**DOI:** 10.3390/ijms27020664

**Published:** 2026-01-09

**Authors:** Hideki Hayashi, Yoshinao Kubo, Yoshimasa Tanaka

**Affiliations:** 1 Center for Medical Innovation, Nagasaki University, Nagasaki 852-8588, Japan; ystanaka@nagasaki-u.ac.jp; 2 Department of Clinical Medicine, Institute of Tropical Medicine, Nagasaki University, Nagasaki 852-8523, Japan

**Keywords:** SARS-CoV-2 infection, host reactions, interferon-α and β, interferon-λ, interferon-γ, cytokines, vaccine, cytokine storm

## Abstract

The COVID-19 pandemic has profoundly affected societies around the world. Although the emergency phase of coronavirus disease 2019 (COVID-19) has ended, the threat it poses remains persistent. This review aims to clarify the mechanisms of SARS-CoV-2 (severe acute respiratory syndrome coronavirus 2) infection to support effective management of the disease. A central focus is the host cellular response to the viral infection, with particular emphasis on the role of cytokines. Cytokines play a dual role in antiviral defense: they contribute to the inhibition of viral replication and facilitate the clearance of pathogens, yet dysregulated cytokine responses can result in severe immunopathology. Interferons (type I, type II, and type III) and other cytokines are pivotal in activating intracellular antiviral mechanisms and in orchestrating the recruitment of immune cells through extracellular signaling. Effective immune responses to viral infections are governed not only by primary immune cells—such as dendritic cells, T lymphocytes, and B lymphocytes—but also by the local cytokine milieu shaped by infected and neighboring cells. Given the presence of endogenous inhibitors and autoantibodies in vivo, it is essential to evaluate the functional activity of cytokines in clinical samples. We propose a novel approach to quantify biologically active cytokine levels.

## 1. The SARS-CoV-2 Life Cycle

**Figure 1:**
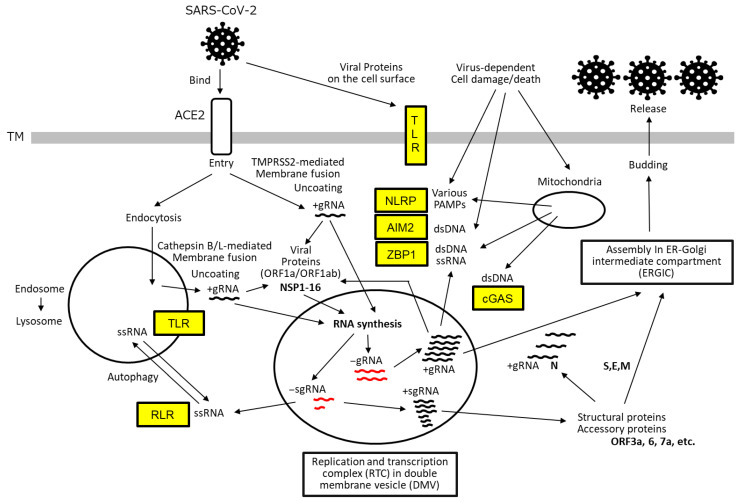
SARS-CoV-2 life cycle and host cellular sensors. SARS-CoV-2 initiates infection by binding to the host ACE2 receptor, followed by membrane fusion and uncoating to release its +gRNA. This process is facilitated by proteolytic cleavage of the viral S protein by TMPRSS at the cell surface or by cathepsin B/L within endolysosomes. The +gRNA is translated by host ribosomes to produce poly-proteins encoded by ORF1a and ORF1ab. These polyproteins are subsequently cleaved by viral proteases—NSP3 (PL^pro^) and NSP5 (3CL^pro^)—to yield mature non-structural proteins (NSP1–NSP16). NSP3, NSP4, and NSP6 coordinate the formation of DMVs, which serve to compartmentalize RTC and shield it from host immune detection. Within the RTC, the viral RdRP composed of NSP7, NSP8, and NSP12 synthesizes full-length gRNA and a series of sgRNAs (shown in red) using the +gRNA as a template. These negative-sense RNAs are then used to generate new +gRNA and +sgRNAs. The +sgRNAs are translated into structural proteins (S, E, M, and N) and accessory proteins (encoded by ORF3a, 6, 7a, etc.). The newly synthesized +gRNA, along with the N protein and other structural components, is transported to ERGIC, where it is assembled into mature virions and subsequently released from the host cell via exocytosis. Throughout the viral life cycle, host cells deploy a range of PRRs, indicated in yellow boxes, to detect viral components. These include TLRs, RLRs, NLRPs, AIM2, and ZBP1. These sensors initiate downstream signaling cascades that contribute to the host immune response.

SARS-CoV-2, *Betacoronavirus pandemicum* is an enveloped, positive-sense, single-stranded RNA virus belonging to the Coronaviridae family [1,2,3,4]. The virion consists of spike (S), envelope (E), membrane (M), and nucleocapsid (N) proteins, and its life cycle is illustrated in Figure 1. Upon entry into the host organism, the innate immune system is rapidly activated [5]. After evading the local host barriers, including the mucosal barrier, SARS-CoV-2 uses its spike protein to bind angiotensin-converting enzyme 2 (ACE2) receptors located on the surface of susceptible cells, thereby facilitating viral entry [1,2,3,4]. Viral entry and uncoating require host proteases to expose the hydrophobic fusion peptide, enabling membrane fusion between the viral envelope and host cell membrane. This process involves transmembrane protease serine 2 (TMPRSS2) for plasma membrane fusion and cathepsin B/L for endolysosomal fusion following endocytosis.

Subsequently, the positive-sense single-stranded genomic RNA (+gRNA) is released into the cytoplasm, where it is translated into polyproteins from open reading frames ORF1a and ORF1ab using the host’s translational machinery. These polyproteins are cleaved by viral proteases—papain-like protease (PL^pro^) and 3C-like protease (3CL^pro^)—to yield non-structural proteins (NSP1–NSP16), which coordinate the formation of double-membrane vesicles (DMVs). These vesicles, derived from the host endoplasmic reticulum (ER) and remodeled by NSP3, NSP4, and NSP6, function to shield the replication–transcription complex (RTC), thereby evading the activation of immune responses such as interferon (IFN) and cytokine production.

Within the RTC, the viral RNA-dependent RNA polymerase (RdRP) synthesizes full-length negative-sense genomic RNA (−gRNA) and a series of negative-sense subgenomic RNAs (−sgRNAs) via discontinuous transcription at transcription regulatory sequences (TRSs) on the +gRNA template. These −gRNA and −sgRNAs then serve as templates for the synthesis of new +gRNA and +sgRNAs, respectively.

The +sgRNAs are translated into structural proteins (spike [S], envelope [E], membrane [M], and nucleocapsid [N] proteins) and interspersed accessory proteins (ORF3a, 6, 7a, etc.). The newly synthesized +gRNA, along with the N protein and other structural components, is transported to the ER-Golgi intermediate compartment (ERGIC), where it is assembled into mature virions. These virions are subsequently released from the host cell via exocytosis (Budding).

## 2. Host Responses to Virus Infection

**Figure 2:**
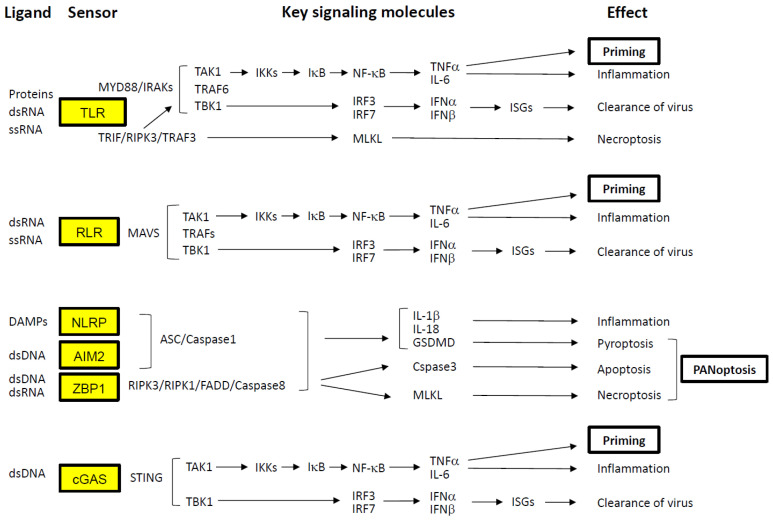
Signaling pathways activated by virus detection. Cellular sensors involved in the recognition of SARS-CoV-2 are highlighted in yellow boxes. PRRs, such as TLRs and RLRs, detect PAMPs derived directly from the virus. In contrast, other PRRs—including NLRPs, AIM2, ZBP1, and cGAS—respond to DAMPs generated by virus-induced cellular injury. These DAMPs include extracellular ATP, elevated intracellular calcium, potassium efflux, ROS, and cytosolic dsDNA. Activation of these sensors initiates downstream signaling cascades that lead to the induction of NF-κB, type I IFNs, and, in some cases, programmed cell death. NF-κB activation promotes the transcription of pro-inflammatory cytokines such as TNF-α and IL-6. TNF-α, in particular, exerts a Priming effect by upregulating the expression of key signaling components, thereby amplifying the immune response. Type I IFNs (e.g., IFN-α and IFN-β) stimulate the expression of ISGs, which possess potent antiviral properties. Concurrently, virus-induced cell death pathways—including necroptosis (mediated by MLKL), pyroptosis (mediated by GSDMD), and apoptosis (mediated by caspase-3)—are often activated simultaneously. This coordinated cell death response is referred to as PANoptosis. Arrows indicate the direction of signaling pathways.

**Figure 3:**
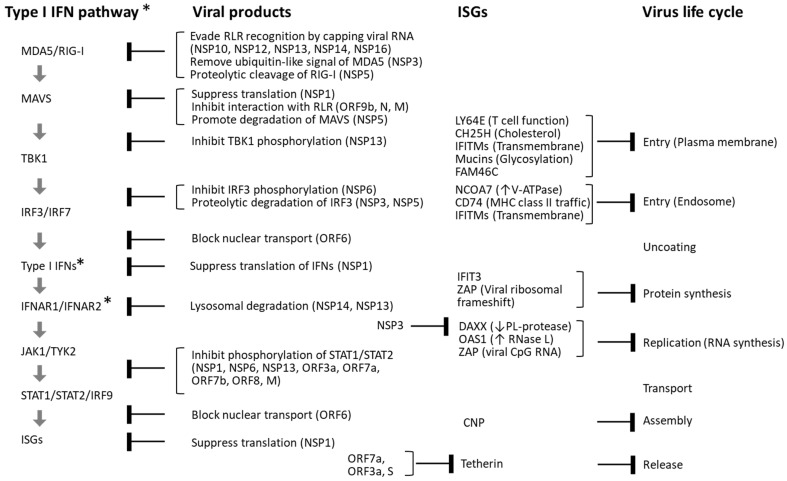
Interference between SARS-CoV-2 and the type I IFN pathway. SARS-CoV-2 infection induces the production of type I IFNs, which in turn stimulate the expression of a wide array of ISGs with antiviral functions. However, SARS-CoV-2 has evolved multiple mechanisms to inhibit this antiviral response. Viral proteins encoded by ORF1a and ORF1ab—NSP1–NSP16—as well as structural proteins (S, E, M, and N) and accessory proteins (ORF3–ORF10) derived from subgenomic RNAs, interfere with various stages of the type I IFN signaling cascade. In addition to disrupting IFN signaling, several viral components—including NSP3, ORF7a, ORF3a, and the S protein—directly suppress ISG functions. Despite these viral evasion strategies, numerous host ISGs have been identified that inhibit SARS-CoV-2 replication at distinct stages of its life cycle. These include LY6E, CH25H, IFITMs, mucins, FAM46C (TENT5C), NCOA7, CD74, IFIT3, DAXX, OAS1, ZAP, CNP, and tetherin (BST2). Blunt arrows in the figure indicate specific steps in the type I IFN signaling pathway and viral life cycle that are inhibited by SARS-CoV-2 proteins and host ISGs. ↑: Activate, ↓: Inhibit. * *The type III IFN pathway utilizes type III IFNs and the IFNLR1/IFN10RB receptor complex instead of IFNAR1/IFNAR2.*

Once the virus reaches the cell surface, host cells activate multiple mechanisms to detect the invasion and initiate defensive responses (Figure 2). One of the earliest responses of the innate immune system in cells expressing viral receptors is the secretion of type I IFNs and pro-inflammatory cytokines [5,6]. These IFNs activate a broad array of IFN-stimulated genes (ISGs), which function to inhibit viral replication and facilitate the activation of the adaptive immune system in concert with other cytokines [7]. However, viruses have evolved sophisticated strategies to evade or suppress these host defenses (Figure 3).

As illustrated in Figure 2, host cells utilize pattern recognition receptors (PRRs) to detect PAMPs, which are conserved molecular signatures of pathogens [8,9,10]. Some Toll-like receptors (TLR2 and TLR4) recognize viral proteins at the cell surface, while cytosolic receptors such as melanoma differentiation-associated protein 5 (MDA5) and retinoic acid-inducible gene I (RIG-I)—collectively known as RIG-I-like receptors (RLRs)—detect viral RNA within the cytoplasm [11]. Endosomal TLRs, including TLR3, TLR7, and TLR8, also respond to viral RNA [6,12,13].

All TLRs except TLR3 signal through the Myddosome complex (comprising MyD88, IRAKs, TRAF6, TAK1, and TBK1) in response to SARS-CoV-2 structural proteins (E and S) and viral RNA. Within this complex, transforming growth factor-β-activated kinase 1 (TAK1) phosphorylates IκB kinases (IKKs), which in turn phosphorylate the inhibitor of NF-κB (IκB), leading to the degradation of IκB. This releases NF-κB (a p50/p65 heterodimer), allowing its translocation to the nucleus and subsequent transcriptional activation of inflammatory cytokines such as TNF-α and IL-6 [14,15,16]. TNF-α plays a pivotal role in Priming the immune response by upregulating genes involved in inflammasome formation in advance (e.g., IL-1β, caspase-1, and NLRP3) and coordinating adaptive immune responses via T and B lymphocytes [17].

Simultaneously, TBK1 phosphorylates IFN regulatory factors 3 and 7 (IRF3/IRF7), which dimerize and translocate to the nucleus to induce type I IFN production [18,19]. These IFNs act in an autocrine and paracrine manner through the IFN-α/β receptor complex (IFNAR1/IFNAR2), stimulating ISG expression to inhibit viral replication and spread. In contrast, TLR3 and TLR4 can also signal through the Triffosome complex (comprising TRIF, RIPK3, TRAF3, TRAF6, TAK1, and TBK1) in response to viral ligands [12,13]. This pathway activates RIPK3 and RIPK1, which phosphorylate mixed lineage kinase domain-like protein (MLKL). Phosphorylated MLKL oligomerizes and integrates into the plasma membrane, forming pores that induce necroptosis—a form of programmed necrotic cell death—alongside TAK1 and TBK1-mediated signaling [12,13,20,21]. In the cytoplasm, RLRs signal through mitochondrial antiviral-signaling protein (MAVS), forming a complex with TAK1 and TBK1 to activate both TNF-α and type I IFN pathways [11].

In addition to PAMPs, damage-associated molecular patterns (DAMPs) are also involved in SARS-CoV-2 infection [22]. The virus-infected cells release or expose danger signals that originate from the cell itself, such as extracellular ATP, elevated intracellular calcium, potassium efflux, reactive oxygen species (ROS), cytosolic dsDNA, and cellular debris. They are recognized by PRRs such as TLRs, NLRs and RLRs, as well as non-PRRs such as channel proteins and GPCRs. Activation of these receptors induces various cellular responses, including proliferation, immune activation, and cell death, by initiating downstream signaling cascades. Notably, various types of cell death caused by SARS-CoV-2 infection are closely related to severe outcomes [8].

Inflammasome-mediated cell death is triggered by the formation of capasse-1, ASC (apoptosis-associated speck-like protein containing a CARD), and other molecules that detect DAPMs [23]. Among NOD-like receptor family pyrin domain-containing proteins (NLRPs), NLRP3 assembles ASC and Caspase-1 in response to dsDNA [8,20,21]. Once activated, caspase-1 cleaves gasdermin D (GSDMD), whose N-terminal fragments oligomerize and form membrane pores, ultimately triggering pyroptotic cell death. dsDNA also activates other inflammasomes containing AIM2 (absent in melanoma 2) and ZBP1 (Z-DNA binding protein 1) [24,25]. ZBP1 recruits RIPK1 and RIPK3 via RIP homotypic interaction motifs (RHIMs), and FADD/caspase-8 via the death domain (DD) of RIPK1 [26,27]. Caspase-8 subsequently activates caspase-3 to induce apoptosis. Interestingly, caspase-8 also inhibits necroptosis by cleaving RIPK1 and RIPK3, which otherwise phosphorylate MLKL to promote necroptosis [10]. When caspase-8 activity is suppressed, RIPK3- and MLKL-mediated necroptosis proceeds. These inflammasome pathways are interconnected and can simultaneously trigger pyroptosis, apoptosis, and necroptosis—a phenomenon termed PANoptosis. While PANoptosis serves to eliminate infected cells and limit viral dissemination, excessive activation can result in severe pathological consequences, including cytokine storm and microvascular coagulation [8,28,29,30].

Cytosolic dsDNA is also sensed by cyclic GMP-AMP synthase (cGAS), which activates the stimulator of interferon genes (STING) pathway, leading to NF-κB activation via TAK1 and type I IFN production via TBK1 [31,32,33].

## 3. Cytokine Storm

### 3.1. Possible Mechanisms of Cytokine Storm

#### 3.1.1. Historical Background

Historically, the pathogenic secretion of cytokines by activated monocytes and T cells has been linked to toxic shock syndrome (TSS), which is triggered by staphylococcal toxins—classified as superantigens [34,35]. Excessive production of inflammatory cytokines, commonly referred to as a cytokine storm or cytokine release syndrome, is frequently induced by anti-CD3 antibody administration, allogeneic transplantation, chimeric antigen receptor (CAR) T cell therapy, or viral infections [29,36,37,38]. In severe cases of SARS-CoV-2 infection, cytokine storm is triggered, marked by elevated levels of IL-1β, IL-2, IL-4, IL-6, IL-10, TNF-α, IFN-γ, GM-CSF, MCP-1, CXCL10, and others, alongside deficiencies in type I and type III IFNs [28,29,30,39,40,41,42,43,44,45]. Although the precise mechanisms underlying cytokine storms remain incompletely understood, several contributing factors have been identified, including high viral loads, dysregulated cytokine signaling pathways, and extensive lytic cell death.

#### 3.1.2. Characteristics of SARS-CoV-2

Compared to the influenza virus, SARS-CoV-2 more readily induces cytokine storms while simultaneously suppressing type I and type III IFN responses [40]. Although these IFNs typically induce a broad spectrum of ISGs that facilitate viral clearance, SARS-CoV-2 encodes multiple proteins that antagonize this pathway at various levels (see Figure 3). For example, viral proteins NSP13, NSP12, NSP14, and NSP16—assisted by the cofactor NSP10—cap the 5′ ends of viral RNA to mimic host mRNA and evade recognition by RLRs [46,47,48]. NSP3, a papain-like protease with deubiquitinase activity, removes ISG15 modifications from MDA5, while NSP5 (3CL^pro^) cleaves RIG-I, disrupting signalosome assembly and TBK1 activation [49,50].

NSP1 promotes the translation of viral positive-sense genomic RNA (+gRNA) while inhibiting host mRNA translation, targeting key components of IFN signaling such as TYK2 and STAT2 [51,52,53]. Additional viral proteins—including NSP9, NSP8, and NSP16—further suppress host protein synthesis by impairing signal recognition and mRNA splicing [51]. NSP6, in conjunction with NSP3 and NSP4, remodels the ER to form DMVs that house the viral RTC and modulate autophagy [54,55,56,57]. NSP6 also inhibits phosphorylation of IRF3, STAT1, and STAT2, thereby attenuating IFN signaling [58]. The accessory protein ORF6 binds to the nuclear pore complex (NPC), blocking nuclear translocation of IRF3 and STAT1 [59,60,61]. Further suppression of ISG activity is mediated by NSP3, ORF7a, ORF3a, and the spike (S) protein [62,63,64,65,66,67].

Host cells, however, deploy additional mechanisms that contribute to the elimination of invading viruses. Loss-of-function studies have identified host restriction factors such as DAXX and mucins [63,66,68], while gain-of-function experiments have revealed antiviral roles for host proteins including LY6E, CH25H, IFITMs, FAM64C, NCOA7, CD74, IFIT3, ZAP, OAS1, CNP, and tetherin, which act at various stages of the viral life cycle [62,66,69,70,71,72,73,74,75,76].

#### 3.1.3. Host Cells vs. SARS-CoV-2 in the Lung

**Figure 4:**
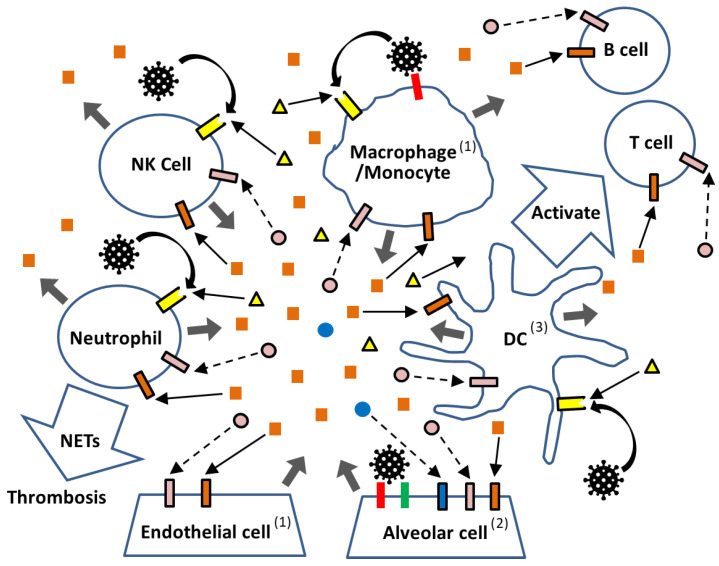
Schematic representation of immunological reactions to SARS-CoV-2 in the lung. In the lung, SARS-CoV-2 virus (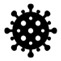) infects alveolar cells via ACE2 (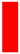), with TMPRSS2 (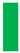) facilitating the viral entry. While PRRs located in the endosome or cytosol (not depicted) detect viral replication products, PRRs (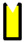) on the cell surface of immune cells (DC, Mnocyte/Macrophage, Neutrophil, and NK cells) recognize the virus as PAMPs without requiring infection, as well as DAMPs (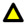) released from virus-damaged cells. Activated immune cells secrete inflammatory cytokines (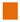), which further stimulate downstream signaling through their respective receptors (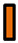). In contrast, diminished type I IFN (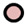) and type III IFN (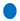) responses fail to deliver sufficient signals through their receptors (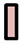 and 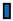). Arrows indicate the direction of signaling (enhanced: 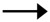 and weakened: 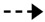). Viral stimulation without infection is shown by curved arrows (↷), and the secretion of inflammatory cytokines and DAMPs is indicated by thick arrows (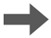). ^(*1)*^ *Some endothelial cells—particularly those in the cardiovascular system—as well as resident macrophages in the lung express ACE2. ^(2)^ Alveolar epithelial cells express ACE2, TMPRSS2, IFNAR, IFNLR, and various PRRs. ^(3)^ Antigen presentation* via *MHC class I and II molecules in DCs and other cells is not depicted.*

As shown in Figure 4, in the lung—the primary target site of SARS-CoV-2—the virus efficiently infects alveolar epithelial cells that express ACE2 and TMPRSS2 [77,78]. It can also infect resident macrophages through ACE2-mediated entry [79,80]. Upon viral entry, intracellular signaling pathways are activated, leading to the production of type I and type III IFNs as well as various inflammatory cytokines (Figure 2). However, SARS-CoV-2 possesses multiple mechanisms to interfere with type I and III IFN production at several steps, enabling the virus to evade host antiviral responses (Figure 3). In many cases, sufficient amounts of type I and III IFNs are produced to activate their respective receptors and induce ISGs, thereby eliminating the virus. This is because nearly all cell types express receptors for type I IFNs, whereas expression of type III IFN receptors is largely restricted to epithelial cells such as alveolar cells [81]. Conversely, if the virus successfully replicates while suppressing IFN-mediated defenses, the newly produced virions not only spread to neighboring cells but also activate intracellular PRRs, and the damaged cells release DAMPs. Both PAMPs and DAMPs stimulate various PRRs located both on the cell surface and within the cytoplasm, triggering the production of inflammatory cytokines such as IL-1β, IL-6, TNF-α, type II IFN (IFN-γ), and IL-10, along with relatively weak type I and III IFN responses. Local immune cells—including DCs, monocytes, and macrophages expressing PRRs—respond to these cytokines and contribute to the development of adaptive immunity by instructing T and B cells, which ultimately eliminate viruses that evade the initial IFN-mediated defense [82,83]. However, when these immune cells have already been activated by PAMPs and DAMPs, they may overreact to inflammatory cytokines, initiating a self-amplifying loop of cytokine production that culminates in a cytokine storm.

#### 3.1.4. Extraordinary Immune Cell Reactions

SARS-CoV-2 drives host cells, including immune cells, to produce extraordinarily high levels of inflammatory cytokines, likely due to its strong ability to suppress IFN signaling, resulting in high viral loads, robust PRR activation, and related downstream events. These excessively produced inflammatory cytokines damage and dysregulate immune cells, as summarized in Table 1. Notably, cytokines such as TNF-α, IL-1β, IL-6, IFN-γ, and IL-10 not only amplify the production of additional cytokines—including themselves—but also play critical roles in regulating immune cell differentiation and function, thereby exacerbating immune dysregulation in severe COVID-19 [82,83,84,85,86,87]. Aberrantly activated macrophages and monocytes are considered major sources of excessive cytokine production, while lymphocytopenia—affecting T cells, B cells, and NK cells—is frequently observed. Meanwhile, type I IFN production by plasmacytoid DCs (pDCs) is reported to be attenuated [88,89].

Overproduced TNF-α and IL-1β exert priming effects by upregulating molecules involved in inflammation and cell death pathways, such as pyroptosis and PANoptosis, respectively [21,28]. Although these cytokines are required for proper DC maturation and for the activation of T cells and B cells, their excessive induction of cell death may contribute to dysregulated adaptive immunity. IL-6 further induces the expression of TNF-α, IFN-γ, and TGF-β, thereby amplifying inflammation and influencing T cell polarization [83,84,85,86]. IFN-γ, known to promote Th1 differentiation, activates various immune cells [83,84,85]. IL-10, on the other hand, is recognized as a key immunosuppressive cytokine predominantly secreted by Tregs [87,90]. Under certain conditions, however, it can also exert immunostimulatory effects on CD8^+^ T cells and B cells. A marked elevation of IL-10 is a hallmark of severe COVID-19, distinguishing it from other betacoronavirus infections such as SARS and MERS [90]. This elevation is not merely a compensatory response to suppress excessive inflammation; rather, its pleiotropic functions may interfere with the establishment of a properly regulated immune response. IL-10 also increases ACE2 expression, and a genetic risk factor has been identified: a polymorphism (rs13050728) dependent readthrough from IFNAR2 into the downstream IL-10 receptor gene, IL10RB, results in the formation of a hybrid receptor (CiDRE) that enhances IL-10 signaling while attenuating type I IFN signaling, thereby worsening COVID-19 severity [91]. Taken together, these findings suggest that extraordinarily high levels of inflammatory cytokines profoundly disrupt proper immune development.

### 3.2. Pathological Consequences

Despite these host defenses, SARS-CoV-2 often achieves high viral titers, leading to elevated levels of proinflammatory cytokines such as TNF-α, IL-1β, IL-6, IL-10, and IFN-γ, alongside suppressed type I and III IFN responses. SARS-CoV-2 has continued to evolve under strong host selection pressures, resulting in variants with increased fitness. Distinct from earlier major variants (Alpha, Beta, Gamma, and Delta), the most recent Omicron variant carries numerous mutations—particularly in the spike protein—that confer increased infectivity and immune evasion [92,93,94,95]. These mutations have shifted the primary target cells from the lung to the upper respiratory tract and attenuated host immune responses, resulting in generally milder clinical outcomes. Omicron variants preferentially utilize endocytosis-dependent entry rather than TMPRSS2-dependent cell surface fusion. Although attenuated, Omicron variants still retain fundamental pathogenic features, including lymphocytopenia affecting both T and B cell populations, as well as hyperactivation of monocytes, macrophages, and neutrophils. This dysregulated cytokine environment likely impairs the development of effective adaptive immunity, as timely and balanced cytokine signaling is essential for orchestrating an appropriate immune response.

Inflammatory cell death—including PANoptosis and NETosis—represents a critical pathological hallmark of severe COVID-19. This process involves widespread death of vascular endothelial and alveolar epithelial cells, accompanied by elevated cytokine release [28,29,30]. Microthrombi frequently contain abundant neutrophils undergoing NETosis, often in close association with platelets. An elevated neutrophil-to-lymphocyte ratio (NLR) and increased formation of neutrophil extracellular traps (NETs) have been linked to severe cases of COVID-19 [28,29,30,96,97,98,99]. Neutrophils are activated through both PRR-dependent and PRR-independent pathways, and overactivated neutrophils produce NETs and undergo NETosis. These NETs activate platelets and, together with inflammatory cytokines, contribute to the induction of PANoptosis or cause damage to alveolar and endothelial cells, ultimately leading to dyspnea and thrombosis [96,97,98,99]. In addition, sera from COVID-19 patients contain pro-NETotic factors such as RANTES (CCL5) and platelet factor 4 (PF4), likely secreted by hyperactivated platelets and their precursor megakaryocytes, which are known to harbor SARS-CoV-2 [99]. Excessive platelet consumption during thrombogenesis may contribute to the development of thrombocytopenia in these patients.

Experimental evidence has demonstrated that combined stimulation with TNF-α and IFN-γ induces PANoptosis in human bone marrow-derived macrophages (BMDMs) in vitro, as well as in the pulmonary and intestinal tissues of transgenic mice expressing human ACE2. Notably, administration of neutralizing antibodies against TNF-α and IFN-γ significantly reduced SARS-CoV-2-associated mortality in vivo [28]. These findings indicate that the complex interplay among cytokine storms, inflammatory cell death, and coagulopathy underscores the therapeutic potential of targeted anti-inflammatory strategies.

## 4. Evaluation of Functional Cytokines

### 4.1. COVID-19 Patients with Anti-IFN Autoantibodies

As illustrated in Figure 3, SARS-CoV-2 attenuates host type I and III IFN responses. Additionally, inborn errors in host immunity influence the outcome of infection. Loss-of-function mutations in type I IFN signaling pathways, including TLR3, IRF3, and IFNAR1, were found in 3.5% of patients with life-threatening COVID-19 [100]. Notably, approximately 10% of all patients with life-threatening COVID-19 had neutralizing autoantibodies against type I IFNs [101,102]. These antibodies, present prior to infection, inhibited type I IFN signaling (e.g., STAT1 phosphorylation and CXCL2 induction) and permitted SARS-CoV-2 replication in vitro. These findings may aid in predicting, preventing, and treating severe cases.

### 4.2. Importance of Evaluating Functional Cytokines

**Figure 5:**
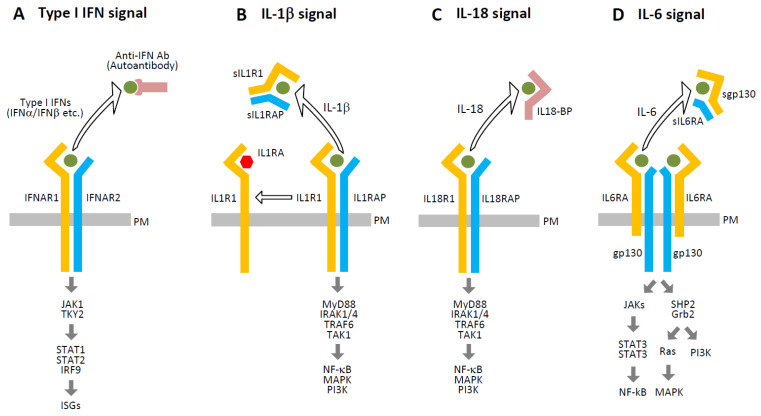
Intrinsic inhibitors of cytokine signaling. This figure illustrates several endogenous mechanisms that attenuate cytokine signaling through competitive inhibition or receptor blockade: (**A**) Autoantibodies targeting type I IFNs (e.g., IFN-α2 or IFN-ω) can impair signaling by preventing their binding to the IFN-α/β receptor complex (IFNAR1/IFNAR2), thereby inhibiting downstream activation. (**B**) IL-1RA binds to IL1R1 and prevents the recruitment of the IL1RAP, which is essential for downstream signaling. Additionally, sIL1R1 and sIL1RAP, generated via proteolytic cleavage or alternative splicing, compete with membrane-bound receptors for IL-1 binding, further reducing signal transduction. (**C**) IL-18BP binds IL-18 with high affinity, sequestering it from the functional IL18R1/IL18RAP receptor complex and thereby inhibiting downstream signaling. (**D**) Soluble IL-6 receptor components (sIL6RA and sgp130) compete with the membrane-bound IL6RA/gp130 receptor complex for IL-6, attenuating the formation of the active signaling complex and reducing IL-6-mediated responses. Open arrows indicate interference caused by the removal of ligands or receptors from their proper signaling complexes, and thick arrows represent the signaling pathways.

In addition to type I IFN antibodies, several molecules are known to inhibit cytokine-receptor interactions, as shown in Figure 5. IL-1 receptor antagonist (IL-1RA) competes with IL-1β for IL1R1 binding but fails to recruit IL1RAP, thereby preventing signaling complex formation [103,104]. Soluble forms of IL1R1 and IL1RAP (sIL1R1 and sIL1RAP), generated via proteolytic cleavage or alternative splicing, further reduce downstream signaling. Similarly, IL-18 binding protein (IL-18BP) binds IL-18 with higher affinity than its receptor complex (IL18R1/IL18RAP), effectively neutralizing its activity [105]. Soluble IL-6 receptor components (sIL-6RA and sgp130) also inhibit IL-6 signaling by competing with membrane-bound receptors [106]. Additionally, molecules released during SARS-CoV-2-induced cell death may interfere with cytokine signaling.

Cytokine levels are commonly measured using ELISA, ELISpot, proteomic profiling, transcriptomic analysis, and single-cell RNA sequencing [28,29,30,39,40,41,42,43,44,45]. However, these methods may not fully reflect cytokine bioactivity due to the aforementioned and other endogenous inhibitors. Since cytokines exert their effects through receptor binding, it is essential to evaluate both cytokine concentrations and the influence of inhibitory molecules.

**Figure 6:**
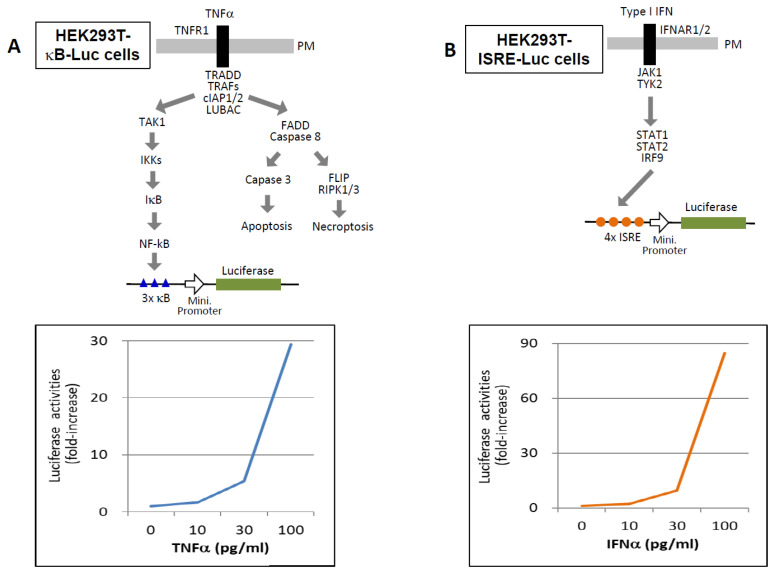
Functional cytokine assays for detecting TNF-α and type I IFNs. (**A**) HEK293T cells stably expressing a luciferase reporter gene under the control of a minimal promoter preceded by three tandem NF-κB response elements (HEK293T-κB-Luc) were used to assess TNF activity. Cells were stimulated with the indicated concentrations of TNF-α, and luciferase activity was measured after 12 h. Results are presented as fold induction relative to the baseline activity of unstimulated cells (medium only), which was set to 1. (**B**) HEK293T cells stably expressing a luciferase reporter gene under the control of a minimal promoter preceded by four tandem interferon-stimulated response elements (ISREs) (HEK293T-ISRE-Luc) were used to evaluate type I IFN activity. Cells were stimulated with the indicated concentrations of IFN-α, and luciferase activity was measured after 12 h. Data are shown as fold induction relative to the unstimulated control (medium only), normalized to a value of 1.

Therefore, it is critical to assess the functional activity of cytokines—specifically, their ability to bind and activate their cognate receptors in the presence of inhibitors. Biological assays using serum samples offer a practical approach for this purpose [107,108,109,110]. For example, HEK293T cells engineered to express a luciferase reporter under the control of an NF-κB-responsive promoter (HEK293T-κB-Luc) respond to TNF-α in a dose-dependent manner (10–100 pg/mL) (Figure 6A). Similarly, HEK293T-ISRE-Luc cells, which express a luciferase gene under the control of an IFN-stimulated response element (ISRE), respond to IFN-α in the same concentration range (Figure 6B). Reported serum concentrations of TNFs and type I IFNs in COVID-19 patients typically range from 1 to 200 pg/mL, although these values can vary depending on the detection method used [28,29,30,39,40,41,42,43,44,45]. These engineered cell lines thus provide a viable platform for assessing functional cytokine activity in patient samples.

**Figure 7:**
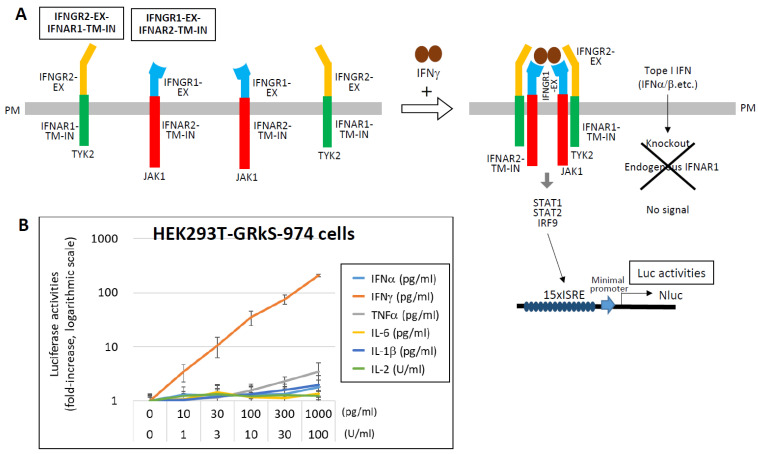
Highly sensitive detection of IFN-γ using chimeric receptor-based reporter cells. (**A**) To enable specific and sensitive detection of IFN-γ, chimeric receptors were engineered by fusing the extracellular domains of IFN-γ receptor subunits (IFNGR2 and IFNGR1) to the transmembrane and intracellular domains of type I interferon receptor subunits (IFNAR1 and IFNAR2), respectively. HEK293T cells stably expressing these chimeric receptors (designated IFNGR2-EX–IFNAR1-TM-IN and IFNGR1-EX–IFNAR2-TM-IN), along with a NLuc luciferase reporter gene driven by a 15 ×ISRE promoter, were established (HEK293T-GRkS-974). In these cells, stimulation with IFN-γ activates the intracellular JAK1/TYK2–STAT1/STAT2–IRF9 signaling cascade, resulting in measurable NLuc luciferase activity. To ensure specificity for IFN-γ, the endogenous IFNAR1 gene was knocked out in these cells, thereby eliminating responsiveness to type I IFNs. (**B**) HEK293T-GRkS-974 cells were stimulated with the indicated concentrations of various cytokines, and NLuc luciferase activity was measured after 12 h. Results are presented as fold induction relative to the unstimulated control (medium only), which was normalized to 1. Data are shown on a logarithmic scale as the mean ± standard deviation from three independent experiments. Data from Ref. [111].

In contrast, the promoter activity of the IFN-γ activation site (GAS) is relatively weak [107,108,109,110]. To enhance sensitivity for IFN-γ detection, a novel system was developed using chimeric receptors. The extracellular domains of IFNGR2 and IFNGR1 were fused to the transmembrane and intracellular domains of IFNAR1 and IFNAR2, respectively (Figure 7) [111]. These chimeric receptors, co-expressed with a 15 × ISRE-driven NLuc reporter in HEK293T cells lacking endogenous IFNAR1, respond specifically to IFN-γ via the Janus kinase 1 (JAK1)/TYK2–STAT1/STAT2–IRF9 signaling axis. The resulting HEK293T-GRkS-974 cells exhibit dose-dependent activation in response to 10–1000 pg/mL of IFN-γ.

Interestingly, cytokine profiles differ markedly between SARS-CoV-2 infection and mRNA vaccination [112]. Following vaccination with an mRNA encoding the S protein, elevated levels of IFN-γ, IL-15, and CXCL10 correlate with robust antibody responses. Serum IFN-γ levels post-vaccination can reach 1–5000 pg/mL, significantly higher than those observed during cytokine storms in COVID-19 (1–200 pg/mL), suggesting effective T cell activation. The relatively low IFN-γ levels in severe COVID-19 may reflect T cell depletion, despite monocyte and macrophage activation. Derivatives of HEK293T-GRkS-974 cells expressing membrane-anchored ZZ domains (ZZ-TM) could be used to identify IFN-γ-producing cells by targeting specific immune cell types with antibodies, thereby distinguishing cytokine storm from normal inflammatory responses.

**Figure 8:**
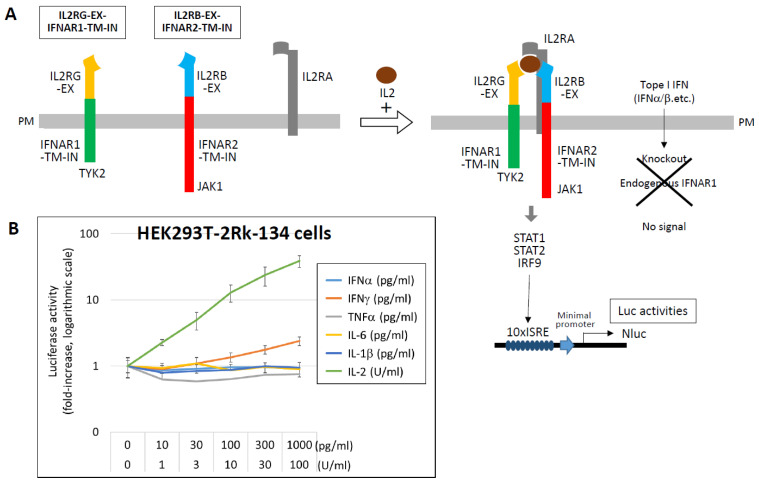
Detection of IL-2 using chimeric receptor-based reporter cells. (**A**) To enable specific detection of IL-2, chimeric receptors were constructed by fusing the extracellular domains of IL-2 receptor subunits IL2RB and IL2RG to the transmembrane and intracellular domains of IFNAR2 and IFNAR1, respectively. HEK293T cells stably expressing these chimeric receptors (IL2RB-EX–IFNAR2-TM-IN and IL2RG-EX–IFNAR1-TM-IN), along with IL2RA and a NLuc luciferase reporter gene driven by a 10 ×ISRE promoter, were established (HEK293T-2Rk-134). Upon stimulation with IL-2, the JAK1/TYK2–STAT1/STAT2–IRF9 signaling cascade is activated, resulting in measurable NLuc luciferase activity. To ensure specificity, the endogenous IFNAR1 gene was knocked out in these cells, eliminating responsiveness to type I IFNs. (**B**) HEK293T-2Rk-134 cells were stimulated with the indicated concentrations of various cytokines, and NLuc luciferase activity was measured after 12 h. Results are presented as fold induction relative to the unstimulated control (medium only), which was normalized to 1. Data are shown on a logarithmic scale as the mean ± standard deviation from three independent experiments. Data from Ref. [111].

For IL-2 detection, chimeric receptors were constructed by fusing the extracellular domains of IL2RB and IL2RG to the transmembrane and intracellular domains of IFNAR2 and IFNAR1, respectively. These were co-expressed with IL2RA and a 10 × ISRE-NLuc reporter in HEK293T cells (HEK293T-2Rk-134), which responded specifically to IL-2 in the range of 1–100 U/mL (Figure 8) [111]. Similar strategies have been employed to develop detection systems for IL-1 and IL-18. These engineered cell lines offer a powerful platform for evaluating functional cytokine activity and may contribute to understanding, predicting, and managing cytokine storm.

## 5. Therapeutic and Preventive Approaches

Recent advances in therapeutic and preventive strategies against SARS-CoV-2 have focused on both viral suppression and modulation of host immune responses [113,114]. A key objective in managing severe COVID-19 is the control of excessive cytokine production and the associated coagulopathy resulting from inflammatory cell death, including NETosis. General anti-inflammatory agents, such as glucocorticoids, have demonstrated efficacy in reducing mortality in severe cases [45].

As illustrated in Figure 1, several viral components serve as targets for antiviral drug development. These include the inhibition of the interaction between the viral S protein and the host ACE2 receptor, as well as the inhibition of virus-specific proteases (PL^pro^ and 3CL^pro^) and RdRP [113,114]. While monoclonal antibodies remain a prominent therapeutic modality, numerous small-molecule inhibitors have also been developed, often with the aid of artificial intelligence to optimize molecular design. To mitigate aberrant host immune responses, immunosuppressive agents targeting key cytokines and signaling pathways have been employed—(1) TNF-α, IL-1β, and IL-6 involved in amplification loops of inflammation; (2) TNF-α, IFN-γ, and IL-1β involved in cell death pathways; and (3) intracellular signaling molecules such as JAK kinases (Figure 2). These interventions aim to reduce the severity of cytokine storm and prevent downstream complications, and inhibition of IL-6, IL-1, and JAK kinases has generally shown clinical benefit in COVID-19 patients [113,114]. However, targeting TNF-α or IFN-γ does not consistently yield beneficial outcomes, as these cytokines possess strong immunostimulatory functions that contribute to viral clearance while also enhancing harmful inflammation, including PANoptosis. In addition, the timely administration of type I and type III IFNs has been reported to be effective in clinical settings [115,116]. Compared to type I IFNs, type III IFNs (IFN-λs) exhibit more targeted activity, acting primarily on epithelial and endothelial cells at sites of infection, while inducing minimal pro-inflammatory responses. Finally, to manage thrombotic complications, anticoagulants or NET inhibitors may be considered, although their benefits are not universal [98,117]. NET production by activated neutrophils and subsequent NETosis can help trap viral particles and limit their spread. However, NETs can also promote thrombosis through platelet activation and contribute to the generation of autoantibodies harmful to the host, ultimately leading to clinical complications [118].

In response to the urgent need for immunization, RNA-based vaccines were the first to be developed and played a pivotal role in controlling the pandemic [119]. To enhance cost-effectiveness and immunogenicity, self-amplifying RNA vaccines have been engineered using NSPs from equine viruses to enable intracellular replication of the RNA payload [120,121,122,123]. Subsequently, protein-based vaccines were developed using innovative antigen presentation strategies to elicit robust neutralizing antibody responses [124,125,126,127,128]. Antigen presentation design is as critical as antigen selection. The receptor-binding domain (RBD) of the SARS-CoV-2 spike protein is the most frequently targeted antigen. Structural features of various proteins have been exploited to enhance immunogenicity. For example, the heptad repeat (HR) region of the spike protein facilitates trimerization, and when fused with the RBD, this trimeric configuration significantly enhances immune responses compared to monomeric RBD [124,125].

Moreover, virus-like particles (VLPs) displaying multivalent antigens have shown superior immunogenicity. A notable example involves the use of engineered aldolase as a scaffold to present 60 copies of the antigen on a dodecahedral structure [126,127]. This fusion protein self-assembles into a multimeric complex, leveraging the trimerization property of aldolase. The 60-mer configuration not only enhances immunogenicity but also offers flexibility in addressing antigenic drift and zoonotic spillover, as it can accommodate multiple antigen variants on a single platform [128].

## 6. Conclusions and Future Perspective

Cellular responses to viral infection, while essential for pathogen clearance, can sometimes result in unintended systemic damage. In this review, we have discussed the early stages of the host–virus interaction, including the development of cytokine storm. To evaluate functional cytokine activity, we propose a novel method utilizing chimeric receptors composed of extracellular domains of interest fused to the transmembrane and intracellular regions of IFNAR1/2.

Following the resolution of the acute phase of the COVID-19 pandemic, a substantial proportion of individuals continue to experience persistent symptoms—commonly referred to as “long COVID” or “PASC” (Post-Acute Sequelae of COVID-19)—that last for months or even years after the initial infection [129,130]. Although cytokine profiles in these patients do not markedly differ from those observed during the acute phase, their levels remain diminished yet persistently elevated, suggesting a smoldering inflammatory state [131]. Although the precise mechanisms underlying long COVID remain unclear, persistent viral reservoirs may continuously drive the production of inflammatory cytokines [132]. Recent studies further suggest that immune dysregulation—particularly the sustained activation of CD8^+^ T cells secreting high levels of IFN-γ—contributes to ongoing respiratory inflammation [133,134]. These findings underscore the importance of regulating aberrantly activated or suppressed cytokine responses and dysfunctional immune cell populations. Such regulation is likely to be critical not only for mitigating acute disease severity but also for preventing or managing long-term sequelae.

## Figures and Tables

**Table 1:** Major immunological effects of the inflammatory cytokines.

Major Producer Cells	Cytokines	Effecter Immune Cells	Biological Effects
Various cells	TNF-α	Neutrophils DC Monocytes/Macrophages NK cells T cells B cells	Activation; Induction of NETosis Maturation Activation; Induction of PANoptosis Activation; Induction of PANoptosis Activation; Exhaustion of CD4^+^T cell Dysregulation of Treg Promotion of Th22 differentiation Activation; Exhaustion of CD8^+^T cell Activation; Induction of PANoptosis
Various cells	IL-6	Monocytes/Macrophages NK cells T cells B cells	Activation Reduction in cytotoxicity Promotion of Th17, Tfh, and Th22 differentiation Inhibition of Treg differentiation Inhibition of Th1 differentiation Reduction in CD8^+^T cell cytotoxic function Activation
Monocytes/Macrophage NK cells DC Endothelial cells	IL-1-β	Neutrophils DC Monocytes/Macrophages NK cells T cells B cells	Activation; NETosis Maturation Activation; Induction of Pyroptosis Activation; Induction of Pyroptosis Promotion of Th17 differentiation Activation; Induction of Pyroptosis in CD8^+^T cell Activation; Induction of Pyroptosis
Monocytes/Macrophages NK cells DC	IFN-γ	DC Monocytes/Macrophages NK cells T cells B cells	Activation; Enhanced antigen presentations Activation Activation Promotion of Th1 differentiation Activation of CD8^+^T cell Inhibit Th2 differentiation Activation
Treg DC Monocytes/Macrophages B cells	IL-10	DC Monocytes/Macrophages NK cells T cells B cells	Inhibition Inhibition Inhibition Inhibition of CD4^+^Th2 and Th17 Activation of CD8^+^T cells Regulation of responses

Major producer cells and effector immune cells for key inflammatory cytokines (TNF-α, IL-6, IL-1β, IFN-γ, and IL-10) are summarized below. Although IL-10 generally exerts inhibitory functions, many cytokines display dual or context-dependent effects depending on the immunological environment.

## Data Availability

No new data were created or analyzed in this study. Data sharing is not applicable to this article.

## Abbreviations

SARS-CoV-2, severe acute respiratory syndrome coronavirus 2; ACE2, angiotensin-converting enzyme 2; TMPRSS, transmembrane protease serine 2; +gRNA, positive-sense single-stranded genomic RNA; −sgRNA, negative-sense subgenomic RNA; ORFs, open reading frames; NSPs, non-structural proteins; PL^pro^, papain-like protease; 3CL^pro^, 3-chymotrypsin-like protease; S, spike; E, envelope; M, membrane; N, nucleocapsid; DMV, double-membrane vesicle; RTC, replication and transcription complex; RdRP, RNA-dependent RNA polymerase; ERGIC, endoplasmic reticulum–Golgi intermediate compartment; TLR, Toll-like receptor; RLR, RIG-I-like receptor; NLRP, NOD-like receptor with pyrin domain; AIM2, absent in melanoma 2; ZBP1, Z-DNA-binding protein 1; ssRNA, single-stranded RNA; cGAS, cyclic GMP-AMP synthase; PRRs, pattern recognition receptors; PAMPs, pathogen-associated molecular patterns; DAMPs, damage-associated molecular patterns; ROS, reactive oxygen species; MyD88, myeloid differentiation primary-response gene 88; IRAK, IL-1 receptor-associated kinases; TRIF, TIR domain-containing adaptor-inducing IFN-β; RIPK, receptor-interacting serine/threonine-protein kinase; TNF, tumor necrosis factor; TRAF, TNF receptor-associated factor; TAK1, transforming growth factor-β-activated kinase 1; TBK1, TANK-binding kinase 1; IKK, IκB kinase; IκB, inhibitor of NF-κB; NF-κB, nuclear factor kappa B; IRF, interferon regulatory factor; ISGs, interferon-stimulated genes; MLKL, mixed lineage kinase domain-like pseudokinase; MAVS, mitochondrial antiviral-signaling protein; ASC, apoptosis-associated speck-like protein containing a CARD; FADD, Fas-associated protein with death domain; GSDMD, gasdermin D; STING, stimulator of interferon genes; MDA5, melanoma differentiation-associated protein 5; RIG-I, retinoic acid-inducible gene I; IFNAR1/2, interferon-α/β receptor subunit 1/2; IFNLR1/IL10RB, IFN-λ receptor 1/IL-10 receptor β-subunit. JAK1, Janus kinase 1; TYK2, tyrosine kinase 2; STAT, signal transducer and activator of transcription; DAXX, death domain-associated protein 6; ZAP, zinc finger antiviral protein; BST2/tetherin, bone marrow stromal antigen 2; IFITMs, interferon-induced transmembrane proteins; CH25H, cholesterol 25-hydroxylase; NCOA7, nuclear receptor coactivator 7; LY6E, lymphocyte antigen 6 complex locus E; OAS1, 2′,5′-oligoadenylate synthetase 1; CD74, invariant chain; FAM46C/TENT5C, terminal nucleotidyltransferase 5C; IFIT3, interferon-induced protein with tetratricopeptide repeats 3; CNP, 2″,3″-cyclic nucleotide 3″ phosphodiesterase; DC, dendritic cell; MHC, Major histocompatibility complex; IL1R1, interleukin-1 receptor type 1; IL1RAP/IL1R2, IL-1 receptor accessory protein; IL-1RA, IL-1 receptor antagonist; sIL1R1, soluble IL-1 receptor type 1; sIL1RAP, soluble IL-1 receptor accessory protein; IL-18BP, IL-18 binding protein; IL18R1, IL-18 receptor type 1; IL18RAP, IL-18 receptor accessory protein; IL6RA, IL-6 receptor α-subunit; gp130, glycoprotein 130; sIL6RA, soluble IL-6 receptor α-subunit; sgp130, soluble glycoprotein 130; SHP2, Src homology 2-containing protein tyrosine phosphatase 2; Grb2, growth factor receptor-bound protein 2; PI3K, phosphatidylinositol 3′-kinase; cIAP, cellular inhibitor of apoptosis; LUBAC, linear ubiquitin chain assembly complex; FLIP, FLICE (FADD-like IL-1β-converting enzyme)-inhibitory protein; 3xκB, three tandemly repeated NF-κB-responsive elements; Min, Minimal; 4xISRE, four tandemly repeated ISRE; IFNGR1, IFN-γ receptor 1; IFNGR2, IFN-γ receptor 2; EX, extracellular domain; TM, transmembrane domain; IN, intracellular domain; Nluc, NanoLuc luciferase; IL2RA, IL-2 receptor subunit α; IL2RB, IL-2 receptor subunit β; IL2RG, IL-2 receptor subunit γ; Treg, regulatory T cell; Th, helper; Tfh, Follicular helper T cell.
